# Interpretation of Drug Interaction Using Systemic and Local Tissue Exposure Changes

**DOI:** 10.3390/pharmaceutics12050417

**Published:** 2020-05-02

**Authors:** Young Hee Choi

**Affiliations:** College of Pharmacy and Integrated Research Institute for Drug Development, Dongguk University_Seoul, 32 Dongguk-lo, Ilsandong-gu, Goyang-si 10326, Gyeonggi-do, Korea; choiyh@dongguk.edu; Tel.: +82-31-961-5212

**Keywords:** drug interaction, pharmacokinetics, tissue-specific, systemic exposure

## Abstract

Systemic exposure of a drug is generally associated with its pharmacodynamic (PD) effect (e.g., efficacy and toxicity). In this regard, the change in area under the plasma concentration-time curve (AUC) of a drug, representing its systemic exposure, has been mainly considered in evaluation of drug-drug interactions (DDIs). Besides the systemic exposure, the drug concentration in the tissues has emerged as a factor to alter the PD effects. In this review, the status of systemic exposure, and/or tissue exposure changes in DDIs, were discussed based on the recent reports dealing with transporters and/or metabolic enzymes mediating DDIs. Particularly, the tissue concentration in the intestine, liver and kidney were referred to as important factors of PK-based DDIs.

## 1. Introduction

Drug-drug interactions (DDIs) are described as the pharmacokinetic (PK) or pharmacodynamic (PD) influence of a perpetrator drug on a victim drug, resulting in an unexpected effect. In practice, DDIs have gained much attention due to their changes of pharmacologic effects (i.e., the loss of efficacy or unintentional toxicity) [1]. The importance of DDIs is well-recognized by the fact that mismanaged DDIs constitute one of the major causes of drug withdrawal from the market (e.g., mibefradil, terfenadine, and cisapride) [2,3,4]. Numerous drug withdrawal cases, as well as serious side-effects caused by DDIs, including PK-based interactions, can be the result of accompanying clinically relevant PD interactions [1,2,3,4]. The DDIs that can change PK profiles involve: (1) Gastrointestinal absorption, (2) protein binding in plasma and/or tissue, (3) carrier-mediated transport across plasma membranes (e.g., hepatic or renal uptake and biliary or urinary secretion), and (4) metabolism. PD interactions, such as acting an agonist or antagonist at the receptor may also increase or decrease the effects of a drug [5].

Since the drug concentration at the target site drives its efficacy and toxicity [6], accurate measurement or prediction of drug concentrations at the target sites is necessary to evaluate DDIs [7,8,9,10,11]. Generally, it is assumed that the plasma drug concentration reflects its concentration in tissue, and drug concentrations in plasma and tissue are mostly thought to be similar. The direct measurement of drug concentration in tissue is practically limited, and the plasma drug concentration has been used as a surrogate for drug concentrations in tissue for PK-PD investigations [11,12,13]. Among possibly changed PK profiles in DDIs, transporter- and/or metabolic enzyme-mediated alteration of plasma concentration of a victim drug by a perpetrator drug is a major proportion to be considered in PK-based DDI evaluations [12,13]. Of course, plasma drug concentrations do not always reflect tissue concentrations [6,14,15,16,17], and moreover, a closer correlation of tissue concentration, not plasma concentration, with PD effects of drugs has emerged (e.g., an increased hepatic concentration of metformin is closely associated with improving its glucose-lowering effect [18,19]; a decreased hepatic concentration of pravastatin is closely associated with a reduction of its lipid-lowering effect [20,21]).

Owing to the accumulation of scientific knowledge for understanding PK-related mechanisms of DDIs and awareness of DDIs, it has become a key issue when assessing DDIs to determine how a victim drug concentration changes at the target (pharmacological action) sites or in the whole body [22,23,24]. Regulatory agencies, such as the United States Food and Drug Administration (FDA) and European Medicines Agency (EMA) have published guidance for drug interaction studies, and they recommend that evaluation of PK-based DDIs be conducted for drugs under development and on the market [22,23]. As a result, detailed information about DDIs via metabolic enzymes and transporters have become available at the time of market approval. Nevertheless, the clinically relevant DDIs, leading to the loss of efficacy or advent of toxicity, have been increasing because polypharmacotherapy is becoming progressively more common. In addition, the underlying mechanisms of DDIs have been variously interpreted, which makes it difficult to explain a correlation of PK and PD changes in DDIs, as well as to predict their clinical relevance [25,26,27,28,29]. In other words, if the alteration of a PD effect is negligible even through a PK change of a victim drug was observed later, the DDI would be ignored at the time. In some cases, if the PK and PD changes of a victim drug are not related, it is likely that the critical underlying mechanism, that triggers a PK change of a victim drug, is mainly associated with a PD effect has not been investigated [25].

Considering that drug concentrations in plasma and tissue are important parameters to investigate DDIs in aspects of PK, as well as PD, we summarized how transporters or metabolic enzymes are involved in the changes of systemic exposure (represented by an area under the plasma concentration versus time curve (AUC)) and/or local tissue concentration of a victim drug in PK-based DDIs.

## 2. Transporter and/or Metabolic Enzymes as Main Determinant Factors in PK-Based DDIs

**Figure 1:**
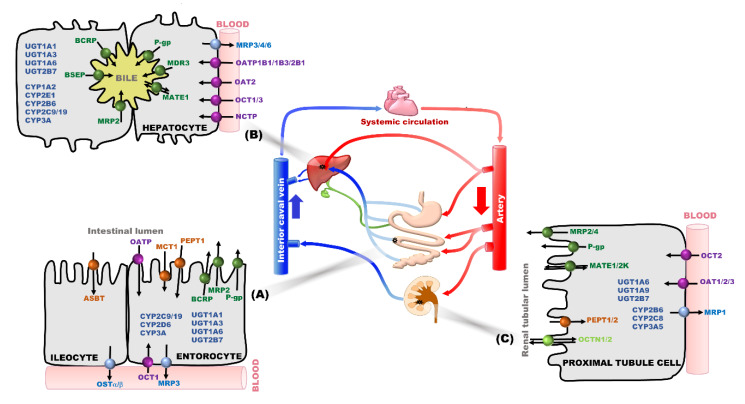
Representative transporters and metabolic enzymes in enterocytes (**A**), hepatocytes (**B**) and renal proximal renal tubules (**C**), which are possibly involved in DDIs.

The individual chemical properties of a victim drug or a perpetrator drug (i.e., molecular size, lipophilicity, ionization, and binding affinity, etc.) can determine its own PK characteristics and affect the PK change of counterpart individual drugs in DDIs [30,31,32]. In addition to the physicochemical properties of drugs, other factors determine the drug concentrations in plasma and tissue, like regulation of inward or outward flow of a drug into blood vessels or tissues by passive diffusion and/or transporters, metabolism by phase I and phase II metabolic enzymes, and/or transporter-mediated excretion (e.g., renal or biliary excretion) [25,33,34]. First of all, a perpetrator drug can affect transporter-mediated uptake and/or efflux of a victim drug across cell membranes of tissues, as well as its excretion from the tissue (e.g., to the blood or outside of the body). Also, a perpetrator drug is able to induce or inhibit metabolic enzymes and consequently change the enzyme-mediated elimination of a victim drug [25,35,36,37]. A transporter-mediated metabolite and/or parent form of a victim drug excretion can be altered by a perpetrator drug as well in DDIs. In other words, the interplay of transporters and metabolic enzymes in enterocytes, hepatocytes, and renal proximal tubules is shown in Figure 1, and their effects on systemic exposure and/or tissue concentration of a victim drug have been increasingly reported [25,33,34,38].

The functions of transporters are to uptake drugs into cells and to export drugs or drug metabolites from the cells. Each transporter has specific expression pattern in individual tissues. In particular, transporters expressed in the small intestine, liver, and kidneys are important for drug disposition and DDIs [39,40,41,42]. We summarized the possible transporters in the human intestine, liver, and kidneys that are able to cause clinically relevant DDIs in Table 1. Considering the absorption, distribution, metabolism and excretion (ADME) process of a drug, efflux transporters such as P-glycoprotein (P-gp; gene symbol ABCB1), the multidrug resistance protein 2 (MRP2; gene symbol ABCC2), and the breast cancer resistance protein (BCRP; gene symbol ABCG2) are localized to the apical membrane of enterocytes (Figure 1A), thereby regulating the bioavailability of orally administered substrate drugs. The concomitantly administered perpetrator drugs inhibited these efflux transporters, resulting in an increase in the bioavailability of the victim drug. In contrast, the bioavailability of the drug was reduced when these efflux transporters were induced by perpetrator drugs. After the absorbed drugs pass through the portal vein and reach the basolateral (i.e., sinusoidal) membrane of hepatocytes (Figure 1B), uptake transporters in the basolateral membrane of hepatocytes mediate the drugs into hepatocytes as the most important site of drug metabolism. For example, organic anion-transporting polypeptides (OATPs) such as OATP1B1 (gene symbol SLCO1B1) and organic cation transporters (OCTs), such as OCT1 (gene symbol SLC22A1), mediate the transport of organic anions and organic cations, respectively. After which efflux transporters localized in the canalicular membrane of hepatocytes [(e.g., P-gp, BCRP, multidrug and toxin extrusion protein 1 (MATE1, gene symbol SLC47A1), multidrug resistance protein 2 (MRP2), and bile salt export pump (BSEP, gene symbol ABCB11)] mediate drug transport into the bile [34,43]. In hepatocytes, the drugs can be metabolized by phase I or phase II metabolic enzymes, and some of them are transported into bile [34,43]. Sometimes, for a small proportion, the efflux of the drug or metabolites across the basolateral membrane into can occur [34].

Drug transporters also play a major role in drug secretion from the renal proximal tubular cells into the urine (Figure 1C). Transporter-mediated uptake across the basolateral membrane of the proximal tubular cells and efflux across the luminal membrane mainly coordinate the renal secretion of a drug, which affects the drug concentration in the blood and kidneys. For the secretion of organic cation drugs, OCT2 (gene symbol SLC22A2), localized in the basolateral membrane uptakes the drug and subsequently, MATE1 and MATE2-K (gene symbol SLC47A2), localized in the luminal membrane, efflux the drug in the proximal tubular cells. Similarly, the uptake and efflux transporters for organic anions are also expressed in the kidney. Alternation of these processes by a concomitantly administered drug leads to reduced or increased renal clearance of the victim drug [43].

After uptake of a victim drug into the tissue, metabolic pathways are involved in PK-based DDIs as an elimination pathway. Cytochrome P450s (CYPs) and UDP-glucuronosyltransferases (UGTs) are the primary metabolic enzymes in phase I and phase II metabolism, as shown in Figure 1 [44,45]. CYP enzymes play a major role in drug elimination through oxidation, reduction, and hydroxylation. They are mainly present on the smooth endoplasmic reticulum and mitochondria of the hepatocytes and small intestinal epithelia, and to a lesser extent in the proximal tubules of the kidneys [46]. Their by-products of metabolism known as metabolites can be either, pharmacologically active or inactive [47]. Among the CYP enzymes in the human liver, CYP3A4 is the most abundant, followed by CYP2E1 and CYP2C9, representing approximately 22.1%, 15.3%, and 14.6% of the total CYP450s (based on protein content), respectively [48], among which the highly expressed CYP isoforms show high potential to cause metabolic DDIs. In addition, drugs or metabolites formed from phase I metabolism are conjugated with a hydrophilic compound with the help of transferase enzymes during the process of phase II metabolism. The most common phase II drug-metabolizing enzymes are UGTs, sulfotransferases (SULTs), *N*-acetyltransferases (NATs), glutathione *S*-transferases (GSTs), thiopurine *S*-methyltransferase (TPMT), and catechol *O*-methyltransferase (COMT), and glucuronidation by UGTs have been reported as major phase II enzyme-mediated DDIs [5,49]. Glucuronide conjugated products are mostly hydrophilic and are readily excreted from the body, mainly through efflux transporters in the liver, intestines, and kidneys (e.g., biliary excretion and renal excretion) [5,49,50]. UGTs are normally highly expressed in the liver and intestine, and their substrates are relatively more overlapping with each other compared to substrate for CYPs. Especially, UGT1A1, 1A3, 1A9, 2A1, or 2B7-mediated DDIs have commonly occurred. These pathways, including interplay of metabolic enzymes and transporters during the ADME of drugs, and any alterations of them may result in changes in the PK and PD of a victim drug [5,45].

## 3. Effects of Transporter- or Metabolic Enzyme Mediated Changes of Systemic Exposure and/or Local Tissue Concentration on PD Effects

Regarding the effects of PK profile changes on DDIs, it is important to consider which PK parameters (or concepts) are primarily or closely associated with PD effects in DDIs [1]. The accurate measurement or prediction of the unbound (free) drug concentration at the target site is essential for evaluating DDIs [6,8,9,10,11]. When considering the protein binding of a drug, it is assumed that only free drugs, not bound to proteins and lipids in the blood and tissues, are metabolized and distributed to target sites where the free drugs exert their pharmacological effects [9,10,58,59]. Protein binding is possibly able to impact PK and PD, driven by the changes of free drug concentrations [58,59]. However, it has been generally accepted that the protein binding change of a victim drug shows little clinical significance in the majority of DDIs cases [58,60].

In the following section, we describe the effects of transporter- and/or metabolic enzymes-mediated DDIs, resulting in changes in systemic exposure or local tissue concentration of a victim drug, which affect PD effects. In particular, since transporters and metabolic enzymes are determinant factors causing DDIs, they are differently expressed and variously interplay together in individual tissues. Therefore, it is difficult to generally explain the systemic exposure and tissue concentration changes with PD effects in accordance with the change (e.g., inhibition or induction) of transporters or metabolic enzymes. Thus, we focused on the change of systemic exposure or tissue concentration in the liver and kidneys of a victim drug along with how their changes affect the PD effects. Several examples are presented in Table 2.

As a major site of drug absorption, the changes in transporters in the apical and basolateral membranes, as well as the metabolic enzymes of enterocytes determine the final amount of drug absorbed into systemic circulation [54,57]. An increase of drug absorption happens due to a drug influx from the intestinal lumen into enterocytes, by an increase of influx transporter or a decrease of efflux transport in the apical membrane, a decrease of drug metabolism in enterocytes, or an increase of drug efflux from enterocytes to blood by an increase of transporter in the basolateral membrane [37,54,57]. Recently, drug catalysis by gut microbiota has emerged as one pathway to regulate drug absorption [61]. A change in drug absorption affects the systemic exposure and intestinal concentration of a drug, and they are generally similar in most cases. In particular, the changed intestinal concentration of a drug can drive the alterations of efficacy and toxicity that occur in the intestine [61,62].

In the liver, the changed final hepatic clearance of a victim drug affects its systemic and local tissue exposure depending on the contribution of the transporter and metabolic enzyme-mediated pathways (i.e., uptake or efflux of a victim drug across the basolateral membrane of hepatocyte, hepatic metabolism, and biliary excretion). There are two major cases. One is that the metabolic plus biliary efflux clearance is a determinant step in drug elimination, so the basolateral uptake becomes the rate-determining step in the hepatic clearance of the drug. In other words, the hepatic clearance is driven mainly by uptake clearance. The inhibition of uptake transporter in the basolateral membrane will increase plasma drug concentrations and systemic exposure of a victim drug, but will not significantly impact the liver AUC of a drug. This may be due to the victim drug being mainly eliminated by the liver [6], and the alteration of basolateral uptake into hepatocytes is the main factor behind the change in the plasma concentration of a victim drug.

The second is that the inhibition of metabolic enzymes or biliary efflux transporters can increase the liver AUC of a victim drug while minimally impacting its plasma AUC [63,64], which may be due to the elimination pathway after uptake into hepatocytes being a determinant factor for final hepatic clearance. In these examples, the changes of plasma and liver concentrations of the drug are not symmetric directions. This asymmetry may lead to misinterpretations of the plasma concentration and/or tissue concentration (e.g., the liver) of a victim drug in DDIs, and in particular, this issue is important to explain the association of PK and PD changes in DDIs [6].

In the kidneys, the alteration of systemic exposure (or plasma concentrations) of victim drugs in renal transporter-mediated DDIs is comparatively moderate compared with those in intestinal or hepatic-transporter mediated DDIs. However, a change in renal clearance of drugs that are mainly eliminated via renal routes can substantially affect the systemic levels of victim drugs, and sometimes these changes are related to efficacy and/or toxicity changes [38,54,65]. Moreover, if the kidneys are the pharmacological target site, a drug concentration change of a victim drug in the kidneys is a critical factor regulating the drug efficacy [66,67,68,69]. From here, we have divided the next section into three subsections as follows: (1) Systemic exposure change of a victim drug having a major PD effect; (2) tissue concentration changes of a victim drug having a major PD effect; and (3) additional factors affecting PD effects along with changes in systemic exposure or tissue concentration of a victim drug.

### 3.1. Changed Systemic Exposure of a Victim Drug Affecting the PD Effect

First of all, it is necessary to clarify the relevance of plasma drug concentration and plasma AUC when assessing DDIs. A plasma AUC alteration implies that the systemic exposure of a victim drug is changed by a perpetrator drug in DDIs, which does not consider the exact time point. Since the AUC is an integrated value from the plasma drug concentration-time curve, the AUC change is not identical to plasma drug concentration changes at all sampling points. If the plasma drug concentration at the specific time points affect PD effects, the plasma drug concentration with the sampling time point or periods should be mentioned to prevent confusion of plasma drug concentrations for plasma AUC. For example, C_max_, the highest plasma drug concentration that is generally related to toxicity, has been used to evaluate or predict a risk of toxicity change in DDIs [24]. Assuming that plasma drug concentrations reflect the efficacy or toxicity of a drug, the systemic exposure based on plasma AUC from time zero to last sampling time (or time infinity) is used as a proper parameter in the evaluation of DDIs by numerous investigators and regulatory regencies (e.g., FDA and EMA) [22,23,24]. The systemic exposure change based on plasma AUC is considered to trigger the drug efficacy or toxicity, and especially, the systemic exposure reflects the duration of drug efficacy: The higher plasma AUC results in a stronger efficacy and/or toxicity [24,25,38]. Of course, there may be an exception, such as onset time or duration time in drug efficacy not being reflected by systemic exposure of a drug (e.g., decreased plasma AUC of clopidogrel is not related to a reduction in anti-coagulant activity; [29]), which is described later in detail.

According to the guidelines for DDI studies suggested by FDA and EMA, the AUC ratio for a victim drug in the presence and absence of a perpetrator drug (AUC_in the presence of a perpetrator_/AUC_in the absence of a perpetrator_) > 5 is determined as a strong PK-based DDI occurrence, because a perpetrator drug may inhibit transporter- or metabolic enzyme-mediated clearance of a victim drug. The AUC ratio for a victim drug in the presence and absence of a perpetrator drug <0.5 is also considered, because a perpetrator drug acts as a strong inducer for transporter- or metabolic enzyme-mediated elimination. Both these strong DDI cases are recommended to be labelled as a victim drug [4,24]. Moreover, the plasma AUC represents systemic exposure of a victim drug, and is used as a critical parameter to adjust dosage regimens to clinical levels. Since the contribution of transporters and metabolic enzymes in the occurrence and/or severity of DDIs is different depending on the individual drug combinations, and because there is a pronounced inter-individual variability in the magnitude of the perpetrator effect, it is hard to predict the DDIs and it is necessary to evaluate them case-by-case.

There are several examples where the systemic exposure of a victim drug affects its pharmacological activity. For example, atorvastatin, a permeable drug (log D_7.4_ = 1.53), is a substrate of the OATP transporter (e.g., OATP1B1, 1B3, and 2B1) and is eliminated primarily by hepatic CYP3A metabolism [52,77,78,97]. In a case of atorvastatin co-administered with cyclosporine as a strong OATP inhibitor and moderate CYP3A inhibitor, a 5- to 16-fold increase of plasma AUC of atorvastatin occurs, and a reduction of the atorvastatin dose is clinically recommended, due to an increased rate of muscle-related toxicity, such as myopathy and rhabdomyolysis [1,43,77,78]. Additional DDI studies of atorvastatin are useful for understanding the underlying mechanism of the AUC change of atorvastatin along with the occurrence of toxicity. Co-administered rifampin, an OATP inhibitor, leads to an increase of plasma AUC of atorvastatin, but co-administered itraconazole, a CYP3A inhibitor, does not cause any change of the plasma AUC of atorvastatin. This result suggests that hepatic uptake of atorvastatin via OATPs is the rate-determining step for the hepatic clearance of atorvastatin [62,79,80], and that the plasma concentration of atorvastatin does not reflect its liver concentration [53]. Although the relationship between an increase of atorvastatin AUC in plasma and its pharmacological activity (e.g., cholesterol lowering activity or toxicity) was not mentioned in this example, it is a good example of the contribution of transporter and metabolic enzymes to changes in plasma profiles of a victim drug that do not affect its tissue concentration (e.g., liver) in DDIs.

Co-administration of cimetidine, a MATE1 and OCT2 inhibitor [51,55,56,100,101], with metformin increases systemic exposure of metformin due to the reduction of renal clearance of metformin via inhibited tubular secretion. The effect of cimetidine on the renal excretion of metformin is time-dependent. In other words, cimetidine inhibited metformin renal secretion up to 6 h after cimetidine co-administration, accompanied by an increased blood lactate/pyruvate ratio from 4 h after cimetidine co-administration and reduced creatinine clearance as a dose and concentration-dependent adverse effect of metformin [100]. This is an example of the increased systemic exposure reflecting a toxicity risk of a victim drug in DDIs.

### 3.2. Changed Local Tissue Concentration of a Victim Drug Affecting the PD Effect

In contrast to the numerous DDIs evaluated by the changes of systemic exposure (i.e., plasma drug concentrations), there are also DDIs that show pharmacological action changes caused by the tissue concentration of a victim drug [53,62,98]. In vivo drug concentration at a target site triggering a PD effect is compared to in vitro efficient concentration, which has been used to explain PK-PD correlations of a victim drug in DDIs [66,98]. In vivo tissue concentration/in vitro half-maximal inhibitory concentration for inhibiting transporter or metabolic enzymes ([I]/IC_50_) or in vivo tissue concentration/in vitro inhibition constant for transporter-or metabolic enzyme ([I]/K_i_) of a perpetrator drug are used to investigate whether the tissue concentration of a perpetrator drug is sufficient to inhibit transporter-or metabolic enzyme-mediated interactions with a victim drug. When the ratio of IC_50_/K_i_ is over 2, the inhibitory interaction of a perpetrator drug to a victim drug can sufficiently happen in vivo in tissue [104,105]. Especially, if there is a tissue lag time relative to peak plasma concentration, the duration of drug efficacy or toxicity should also be considered [6,106,107].

After a drug is absorbed into blood and delivered to the tissue, the drug concentration in the tissue is the sum of the net membrane permeation by influx and efflux across the membrane and the intrinsic clearance by metabolism and/or excretion (e.g., biliary excretion in the liver and renal excretion in the kidneys) [33,34,108,109]. Although membrane permeation and intrinsic clearance are variable in individual tissues, drug influx consists of transporter-mediated uptake and passive diffusion from blood to tissue, as well as transporter-mediated active efflux. After the sum of membrane permeation, a drug is exposed to metabolic enzymes and/or excretion pathways within the localized tissue, which determines the drug concentration in the tissue, as well as the plasma drug concentration as systemic exposure of a drug.

When specific tissues are the pharmacological target sites to show drug efficacy and/or toxicity, drug concentration changes in these tissues strongly affect the PD changes in DDIs. The tissue concentration of a victim drug does not necessarily change in parallel to plasma AUC of a victim drug. Thus, it is necessary to choose a tissue to evaluate DDIs based on the pharmacological mechanism and major organ regulating drug disposition. The liver and/or kidneys are frequently involved in drug disposition pathways, but the pharmacological target organs absolutely depend on the individual drugs used. Time-dependent changes of drug concentrations in tissues also need to be considered, especially when onset or duration time is an important factor for drug efficacy [38].

There are several cases showing no change of AUC of a victim drug, even though there is a change of tissue drug concentration, which has been named a silent interaction [63,98]. In the case of atorvastatin with metformin co-administration as a silent interaction, co-administered metformin reduces the biliary excretion of atorvastatin by MRP2 inhibition, increasing the atorvastatin concentration in the liver without changes in total clearance or systemic exposure of atorvastatin [98]. Shin et al. [98] concluded that the increased atorvastatin concentration in the liver might consequently improve the lipid-lowering effect of atorvastatin as in similar DDI studies of statins and metformin [110,111].

In the case of rosuvastatin, the inhibition of OATP uptake by rifampin leads to an increase of blood AUC of rosuvastatin without any significant change to the hepatic AUC of rosuvastatin in rats [87]. Similarly, PBPK analysis of rosuvastatin in humans illustrated that the predicted liver concentrations of rosuvastatin are not significantly impacted. Moreover, the OATP1B1 polymorphism affects the plasma concentrations of rosuvastatin without affecting its PD response [112]. These cases demonstrate that the plasma exposure changes do not reflect liver exposure changes without any relevant PD effects. This is because the rate-determining step in the hepatic clearance of rosuvastatin is uptake clearance via OATPs, even though rosuvastatin is also excreted into bile via MRP2/BCRP [74,75,86,87,88,112].

The case of metformin, an anti-diabetic agent, is an interesting example. Due to metformin’s high pKa (most portions are positively charged at pH 7.4) and its negative logP value, the passive diffusion of metformin through cellular membranes is minor. Therefore, transporters are pivotal for metformin permeation through cellular membranes. In the intestine, apical OCT3 and basolateral OCT1 primarily mediate metformin absorption. In the liver, basolateral OCT1 and OCT3 uptake metformin from the sinusoidal blood into hepatocytes, whereas apical MATE1 is thought to efflux metformin into the bile. In renal proximal tubule cells, OCT2, MATE1, and MATE2-K are essential for the renal tubular secretory system of metformin [56]. The pharmacological action site of metformin is the liver, but renal excretion as an unchanged form, is the main elimination route of metformin, which primarily contributes to determining the systemic exposure of metformin along with intestinal absorption. Metformin disposition changes, such as in plasma and tissue concentration with relevant pharmacological effect alterations is determined by OCT and MATE mediated metformin transport. This has been proven based on studies conducted of metformin administration to patients with OCTs and MATEs genetic polymorphisms, as well as OCTs and MATEs knock-out mice [101,113,114,115]. With regards to DDIs of metformin, Cho et al. [19] reported that rifampin significantly enhanced the glucose-lowering action of metformin (54.5% of AUC_glucose_ with *P* = 0.020) due to the increased mRNA level of OCT1 by rifampin probably enhancing hepatic uptake of metformin. Conversely, co-administration of an OCT1 inhibitor, verapamil, with metformin decreased the glucose-lowering effect of metformin without increases of systemic exposure of metformin in healthy participants [18]. In mice, co-infusion of cimetidine increases metformin concentrations in the liver and kidneys, due to the inhibition of mMate1-mediated metformin export to biliary excretion and renal excretion, respectively [51,55,56], suggesting that cimetidine exerts MATE1 inhibition-mediated interaction with metformin. In another mouse study, co-administration of metformin with pyrimethamine, a MATE inhibitor, also resulted in a ~2.5-fold increase in liver AUC of metformin compared with controls (i.e., metformin alone administration [101,102,114,115]). These examples indicate that the inhibition or induction of transporters primarily mediating drug disposition to pharmacological target tissue has a strong potential to alter the efficacy of the drug, regardless of the systemic exposure, such as plasma concentration.

Although we focused on DDIs in this review, similar phenomena have been observed in herb-drug interactions. Han et al. [67] reported that the glucose tolerance activity of metformin was enhanced without a change of metformin plasma concentration, because the metformin concentration in the liver increased as a result of a reduction of mate1-mediated biliary excretion of metformin in rats simultaneously treated with metformin and *Lonicera japonica* extract. Considering that renal clearance is the main route of metformin’s elimination, only hepatic transporter-mediated interactions of metformin will impact its hepatic concentration, and therefore, affect the PD effect without the alteration of its plasma concentration.

Interestingly, there were several cases where the systemic exposure and tissue exposure of a drug changed in the opposite direction (e.g., increase of systemic exposure and decrease of hepatic exposure). For example, when paroxetine is co-administered with pravastatin in rats, paroxetine increased the systemic exposure and decreased the liver exposure of pravastatin by the combined effects of an increase in intestinal absorption and a decrease in hepatic uptake of pravastatin via Oatp2 inhibition as well as increased biliary excretion via Mrp2 inhibition [96]. The reduced hepatic exposure of pravastatin had a trend to weaken the lipid-lowing effect of pravastatin in diabetic rats [20,21] in spite of the increased systemic exposure of pravastatin. In a case of herb-drug interactions, You et al. [66] reported that the AUC of metformin was increased due to the decrease of oct2-mediated renal excretion of metformin, and thus, the metformin concentration in the kidneys increased due to the increase in oct1-mediated renal uptake of metformin along with the enhancement of its glucose-lowering effect in rats with 28-day co-treatment of metformin and *Houttuynia cordata* (*H. Cordata*) extract. Considering that metformin’s pharmacological action site is the liver, the enhanced glucose-lowering effect is probably due to increased hepatic uptake of metformin by the *H. Cordata* extract. In spite of an increase of metformin’s systemic exposure, any toxicity of metformin identified as renal dysfunction and lactic acidosis were not observed. Thus, the metformin-*H. Cordata* extract combination case can be included as an example of a local tissue concentration change more strongly affecting the PD effect.

### 3.3. Additional Factors Affecting PD Effects with Changes of Systemic Exposure or Local Tissue Concentration of a Victim Drug

Additional underlying mechanisms can also cause PD alterations in DDIs. The first case represents how a PK change of an active metabolite can affect the PD effect in DDIs. In the case of clopidogrel with co-administration of aspirin, the systemic exposure of clopidogrel is reduced due to the intestinal P-gp induction lowering the bioavailability of clopidogrel, but the relative platelet inhibition effect of clopidogrel is not changed [29]. Since co-administered aspirin increases clopidogrel metabolism via CYP2C19 and the AUC of the active thiol metabolite, H4, of clopidogrel is consequently increased, the reduced platelet inhibition effect due to the reduced AUC of clopidogrel might be compensated for by an increase of H4′s AUC [29]. This example indicates that the systemic exposure of a parent drug is not always able to explain the alteration of PD effect, and the systemic exposure of active metabolites can also cause PD changes. Thus, the systemic exposure of a parent drug, as well as active metabolites need to be considered together, especially when predicting pharmacological effects.

The second case is that the co-administration interval between a victim drug and a perpetrator drug can determine the PD effect. Co-administration of nuciferine, as a potential inhibitor of OCT1 and MATE, time-dependently reduced the glucose-lowering effect of metformin and the hepatic metformin concentration. The hepatic metformin concentration was increased until 1 h after nuciferine administration, which subsequently enhanced the glucose-lowering effect of metformin only during the 2.5–4 h after nuciferine co-administration [99]. If the metformin concentration in the liver is measured at longer time intervals after nuciferine co-administration, the increased hepatic metformin concentration, due to the increased OCT1 and reduced MATE1 transport activity for metformin movement by nuciferine would not be detected, and the alteration of the glucose-lowering effect would also be in the same direction. This example indicates that the onset and duration time of pharmacological action is one factor to be considered to evaluate the PK and PD changes in DDIs.

## 4. Challenging Experimental Approaches for Exploring the Transporter- or Metabolic Enzyme- Mediated Ddis

It is necessary to explore the expression or activity changes of transporters- or metabolic enzymes responsible for DDI occurrences. Even though the guidance from regulatory agents (e.g., EMA and FDA), labeling recommendations, and the reported references have informed the DDI potential, general in vitro and in vivo methods, as well as clinically designed approaches have been applied in DDI evaluations [22,23,24,25,37] and elsewhere, the inconsistency between the clinical relevance and the accumulated information (or experimental results) is still present [66,67,79,98] and elsewhere. Besides the conventional tools (e.g., western blot analysis, q-PCR analysis, permeability test, etc.) to measure the protein and mRNA expression or activity of transporters or metabolic enzymes in tissues, require a guess as to the target causing PK alteration. Therefore, the proteomic quantification methods with “total protein approach” (TPA) for transporters and metabolic enzymes in liver or intestines have been attempted [116]. The TPA-based quantification of transporters and enzymes in human liver tissue samples using liquid chromatography-tandem mass spectrometry can detect their protein expression levels, which could correlate with the results from the targeted proteomic quantitation. This challenging TPA-based quantification can identify as many proteins as possible in a small volume of the same sample and does not require standards [116,117]. In addition, unexpected interplay of transporters or metabolic enzymes in DDI events can be detected by TPA-based quantification approach.

As an experimental approach to investigate the tissue exposure, microdialysis technique in tissue distribution studies has recently been introduced, making it possible to measure the drug concentration at multiple time points in one living animal [118,119]. Animals are sacrificed at each sampling point using the classical experimental approaches to measure drug concentration in tissues, and at least four sampling time points are recommended to explain the tissue distribution pattern changes in DDIs, considering C_max_ and terminal half-life [90,120]. Additionally, (1) composition of a victim drug and a perpetrator drug, and (2) the dosage regimen are suggested to be considered in designing in vivo conventional PK experiments. A perpetrator drug can be chosen as an index substrate, inhibitor or inducer of the transporter or metabolic enzyme responsible for the PK of a victim drug, or as a highly recommended drug in clinical combination therapies [3,25,28]. The dosage regimens, including doses, treatment period (i.e., single or multiple treatments), and drug-dosing schedule of a victim drug and a perpetrator drug, can result in different DDI events [6,22,23,24,25,66,67,121].

## 5. Concluding Remarks

Evaluations of DDIs are a crucial issue for drug efficacy and safety. Although methodologies to evaluate DDIs and information about mechanisms of PK-based DDIs have greatly advanced, the incidences and severity of DDIs still remain high in clinical cases. Moreover, previously unknown DDIs have been revealed and their mechanisms have been suggested in clinical DDI studies, but most of them have then been confirmed by, for example, in vitro studies through a reverse translation process. Since clinical DDI studies may not cover the numerous combinations and various factors that cause these outcomes, it is necessary to interpret the PK and PD data to provide a comprehensive understanding of DDIs with clinical relevance. Although there is currently no optimal way to study DDIs, its evaluation needs to be based on interpretation of the available data about PK-based DDIs, which can ensure the safety and maximal usefulness of DDI studies.

## Figures and Tables

**Table 1:** Characteristics of transporters mediating the occurrence of clinically relevant DDIs.

Protein	Location	Direction	Ref.
P-gp (MDR1)	Apical membrane in enterocyte	efflux	[43,51]
	Canalicular membrane in hepatocyte	efflux	
	^1^ Luminal membrane in renal proximal tubule cell	efflux	
MDR3	Canalicular membrane in hepatocyte	efflux	[43]
BSEP	Canalicular membrane in hepatocyte	efflux	[43]
BCRP	Apical membrane in enterocyte	efflux	[43]
	Canalicular membrane in hepatocyte	efflux	
	^1^ Luminal membrane in renal proximal tubule cell	efflux	
MRP1	Basolateral membrane in renal proximal tubule cell	efflux	[6,43]
MRP2	Apical membrane in enterocyte	efflux	
	Canalicular membrane in hepatocyte	efflux	
	^1^ Luminal membrane in renal proximal tubule cell	efflux	
MRP3	Basolateral membrane in enterocyte	uptake	
	Basolateral membrane in hepatocyte	efflux	
	Basolateral membrane in renal proximal tubule cell	efflux	
MRP4	Basolateral membrane in hepatocyte	efflux	[38,43]
	^1^ Luminal membrane in renal proximal tubule cell	efflux	
MRP5,6	Basolateral membrane in hepatocyte	efflux	[43]
OATP1B1	Basolateral membrane in enterocyte	uptake	[43,52]
	Basolateral membrane in hepatocyte	uptake	
OATP1B3	Basolateral membrane in enterocyte	uptake	
	Basolateral membrane in hepatocyte	uptake	
OATP2B1	Basolateral membrane in enterocyte	uptake	[43,53]
	Basolateral membrane in hepatocyte	uptake	
OAT1	Basolateral membrane in renal proximal tubule cell	uptake	[43]
OAT2	Basolateral membrane in hepatocyte	uptake	[38,43]
	Basolateral membrane in renal proximal tubule cell	uptake	
OAT3	Basolateral membrane in renal proximal tubule cell	uptake	[43]
OAT4	^1^ Luminal membrane in renal proximal tubule cell	efflux/uptake ^2^	
OCT1	Basolateral membrane in enterocyte	uptake	[43,54,55,56]
	Basolateral membrane in hepatocyte	uptake	
OCT2	Basolateral membrane in renal proximal tubule cell	uptake	
OCT3	Basolateral membrane in enterocyte	uptake	
	Canalicular membrane in hepatocyte	efflux/uptake	
	Basolateral membrane in renal proximal tubule cell	uptake	
MATE1	Canalicular membrane in hepatocyte	efflux/uptake	[43,51]
	^1^ Luminal membrane in renal proximal tubule cell	efflux/uptake ^2^	
MATE2-K	^1^ Luminal membrane in renal proximal tubule cell	efflux/uptake ^2^	
PEPT1	Apical membrane in enterocyte	uptake	[43,57]
	^1^ Luminal membrane in renal proximal tubule cell	uptake ^2^	
PEPT2	^1^ Luminal membrane in renal proximal tubule cell	uptake ^2^	

^1^ Luminal membrane means apical membrane. ^2^ Uptake in apical membrane in kidneys represents a reabsorption pathway.

**Table 2:** Examples of transporter- or metabolic enzyme-mediated DDIs.

Victim Drug	Perpetrator Drug	Underlying Mechanism ^1^	PK Change of a Victim Drug	PD Change of a Victim Drug	Ref.
Apixaban	Ketoconazole	(−) P-gp in enterocyte	AUC↑	ADR↑ (bleeding risk)	[70]
Dabigatran	Rifampin	(+) P-gp in enterocyte	AUC↓	TR 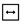 , safety 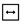	[71]
Digoxin	Rifampin	(+) P-gp in enterocyte	AUC↓	TR↓	[72]
Loperamide	Quinidine	(−) P-gp in enterocyte or brain	AUC↑	ADR↑(respiratory depression) by P-gp inhibition in brain (not enterocyte)	[73]
Rosuvastatin	Eltrombopag, Fostamatinib	(−) BCRP in enterocyte	AUC↑	ADR↑ (myopathy), TR↑	[43,74,75]
Clopidogrel	Aspirin	(+) P-gp in enterocyte (+) CYP2C9 in hepatocyte	AUC↓, F↓, H4 (active metabolite)↑	Platelet inhibition effect 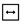	[29]
Digoxin	Clarithromycin	(−) P-gp in enterocyte (−) CYP2D6 or 3A4 inhibition in hepatocyte	AUC↑, CL↓, CL_R_↓ (by non-glomerular renal clearance)	ADR↑ (digoxin toxicity)	[76]
**Atorvastatin**	**Cyclosporine**	(−) OATP1B1, 1B3, 2B1 in hepatocyte (−) CYP3A in hepatocyte	AUC↑, hepatic uptake 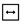	Muscle-related toxicity↑	[1,43,77,78]
	**Itraconazole**	(−) CYP3A in hepatocyte	AUC↑, hepatic uptake 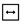	-	[62,79,80]
Bosentan	Clarithromycin	(−) OATP1B1, 1B3 in hepatocyte	AUC↑	ADR↑ (cholestatic liver injury)	[81,82]
Pitavastatin	Cyclosporine, rifampin	(−) OATP1B1, 1B3, 2B1 in hepatocyte	AUC↑	ADR↑	[83,84]
Atrovastatin, simvastatin	Itraconazole, mibefradil, verapamil	(−) CYP3A4 in hepatocyte	AUC↑, C_max_↑	ADR↑ (myopathy, fatal rhadomyolysis)	[85]
Atrovastatin, pravastatin, simvastatin	Clarithromycin	(−) OATP1B1, 1B3, 2B1 in hepatocyte (−) CYP3A4 in hepatocyte	AUC↑, C_max_↑	ADR↑ (myopathy, fatal rhadomyolysis)	[85]
Rosuvastatin	Cyclosporine	(−) OATP1B1, 1B3, 2B1 in hepatocyte	AUC↑, hepatic cons ^2^ 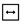	ADR↑	[86,87]
	Gemfibrozil	(−) OATP2B1 in hepatocyte	AUC↑, hepatic cons ^2^ 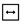	ADR↑	[87,88]
Simvastatin	Cyclosporine	(−) OATP1B1 in hepatocyte (−) CYP3A4 in hepatocyte	AUC↑	ADR↑ (myopathy)	[43]
Adefovir	Probenecid	(−) OAT1 in proximal tubule cell	AUC**↑**, CL_R_↓	ADR↑ (nephrotoxicity)	[43,89]
Benzylpenicillin	Probenecid	(−) OAT3 in proximal tubule cell	AUC↑, CL_R_↓	ADR↑	[89]
Digoxin	Quinidine	(−) CYP2D6 or 3A4 inhibition in hepatocyte (−) P-gp in proximal tubule cell	AUC↑, CL↓, CL_NR_↓, CL_R_↓	ADR↑ (digoxin toxicity)	[83,90]
Lamivudine	Trimethoprim /sulfamethoxazole	(−) OCT2, MATE1, MATE2-K in proximal tubule cell	AUC↑, CL_R_↓	ADR↑ (hepatotoxicity)	[91,92]
Metformin	Trimethoprim	(−) OCT2, MATE1 in proximal tubule cell	C_max_↑, AUC↑, CL/F↓, CL_R_↓	ADR↑ (plasma lactate↑, lactic acidosis especially in renal dysfunction patients)	[93]
		(−) OCT2, MATE1, MATE2-K in proximal tubule cell	C_max_↑, AUC↑, CL_R_↓	ADR↑ (plasma lactate↑, lactic acidosis)	[94]
	Dolutegravir	(−) OCT2 in proximal tubule cell	C_max_↑, AUC↑	ADR↑ (plasma lactate↑, lactic acidosis)	[95]
**Pravastatin**	**Paroxetine**	(−) Mrp2 in enterocyte; (−) Oatp2 in enterocyte/hepatocyte	Intestinal absorption↑, AUC↑, hepatic uptake↓, hepatic cons↓	Lipid-lowing effect ↓	[20,21,96]
Atorvastatin	Rifampin	(−) OATPs in hepatocyte	AUC↑, hepatic uptake↓	Lipid-lowing effect↓	[52,53,79,97]
**Atorvastatin**	**Metformin**	(−) MRP2 in hepatocyte	Biliary excretion↓, hepatic cons ^2^↑	Lipid-lowing effect↑	[98]
**Metformin**	**Rifampin**	(+) mRNA of OCT1 in blood cells	AUC↑ (probable hepatic cons ^2^↑)	Glucose-lowering effect↑	[19]
Metformin	*Lonicera japonica* extract	(−) MATE1 in hepatocyte	AUC 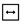 , hepatic cons ^2^↑	Glucose tolerance effect↑	[67]
Metformin	Nuciferine	(−) OCT1 and MATE1 in hepatocyte	Hepatic cons ^2^↓	Glucose-lowering effect↓	[99]
**Rosuvastatin**	**Rifampin**	(−) OATP1B1, 1B3, 2B1 in hepatocyte	AUC↑, hepatic cons ^2^↓, renal cons ^2^↓, hepatic biliary excretion 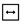	ADR↑	[87]
Metformin	*Houttuynia cordata* extract	(−) MATE1 in hepatocyte (−) OCT2 in proximal tubule cell	AUC↑, CL_R_↓, hepatic cons ^2^↑	Glucose tolerance effect↑	[66]
**Metformin**	**Cimetidine**	(−) MATE1 in hepatocyte; (−) MATE1, MATE2-K in proximal tubule cell	AUC↑, hepatic cons ^2^↑, (biliary excretion↓), renal cons ^2^↑, CL_R_↓	Glucose-lowering effect↑	[51,55,56,100,101]
Metformin	Pyrimethamine	(−) MATE1 in hepatocyte; (−) MATE1 in proximal renal tubule	AUC↑, C_max_↑, CL_R_↓, CL_CR_↓, S_CR_^3^↑, hepatic cons ^2^↑	Glucose lowering effect↑	[101,102,103]

The DDI cases mentioned in the main text is marked by bold style. ^1^ (−) and (+) present inhibition and induction, respectively. ^2^ Cons refers to concentration. ^3^ S_CR_ refers to a serum creatinine level.
